# Study on the combination of lime and lignin in the improvement of mechanical properties of loess in Ili area, China

**DOI:** 10.1371/journal.pone.0341196

**Published:** 2026-02-12

**Authors:** Shichuan Liang, Zizhao Zhang, Xukun Hu, Junyu Jia, Songyuan Lv

**Affiliations:** 1 College of Geology and Mining Engineering, Xinjiang University, Urumqi, China; 2 Xinjiang Uygur Autonomous Region Institute of Geological Environment Monitoring, Urumqi, China; 3 State Key Laboratory for Geomechanics and Deep Underground Engineering, Xinjiang University, Urum, Xinjiang, China; University of Sharjah, UNITED ARAB EMIRATES

## Abstract

Conventional techniques incorporating either inorganic or organic modifiers alone are insufficient to effectively improve loess roadbeds in practice, thus hindering the development of infrastructure. This study proposes the use of lime-lignin as a modifier for loess improvement. Various mixing samples with different additive dosages (0% lime: 0% lignin, 2% lime: 1% lignin, 4% lime: 2% lignin, 6% lime: 3% lignin, and 8% lime: 4% lignin) were prepared and tested in laboratory experiments and numerical simulations to investigate the mechanical properties of the amended loess. Results showed that the compressive and shear strengths of the modified loess initially increased but then decreased with higher lime and lignin contents, with the most optimal improvement achieved at a lime-to-lignin ratio of 2%:1%. The maximum axial stress in the improved loess is achieved with a lime-to-lignin dosage of 2%:1%, the compressive strength of loess increased by 73.97%.The parameters cohesion (c) and angle of internal friction (φ) reached their maximum values at 2%:1% lime and lignin dosing, with maximum values of 208.35 kPa and 24.23°, which were about 149% higher than that of the natural loess.The numerical simulations validated the laboratory results, demonstrating reduced roadbed settlements and improved stress transfer mechanisms. The formation of a complex three-dimensional network structure by lignin fibers and a cement-soil skeleton by lime hydrate and soil particles collectively enhanced the strength of the soil matrix. The findings of this study provide valuable theoretical references for enhancing loess subgrades.

## 1. Introduction

Loess is widely distributed around the world, encompassing regions in East Asia, Central Asia, Central Europe, the United States, northern Russia, interior Alaska, and various parts of South America [1]. In China, approximately 6.6% of the land area is blanketed by loess [2]. Loess is characterized by its loose structure, high porosity, and sensitivity to moisture [3–5]. Therefore, in conditions of sustained rainfall the structural strength of the soil undergoes a notable reduction which leads to a rapid decrease in both mechanical strength and deformation modulus [6–11]. Consequently, soil collapse will result in uneven settlement within loess roadbeds. To ensure the safe construction and operation of projects in these areas, it is imperative to implement measures aimed at enhancing the engineering geological properties of loess [12–15].

According to existing research, the treatment of loess-based foundations or roadbeds encompasses various measures, including compaction, replacement, and composite foundation techniques [16]. In the field of engineering, methods for enhancing highway foundation soils can be categorized into three primary approaches: physical, chemical, and comprehensive improvement strategies [17]. The physical improvement primarily involves the addition of geotextile fabrics [18] and polymer fibers [19] to the soil or modifying particle grading by incorporating materials such as slag, sand, and gravel to bolster the soil’s physical and strength properties [20]. Chemical enhancement techniques usually include introducing materials into the soil, such as cement [21], lime [22], or chemical solutions [23]. Chemical reactions between these additives and soil particles create new substances with enhanced strength properties, thereby improving the overall strength and stability of the soil. Comprehensive amendment techniques are the combination of both physical and chemical amendments [24]. In addition, in recent years, there have been more and more studies on biopolymers. The research status of biopolymers shows the distinctive characteristics of high performance, intelligent function and full-cycle greenization [25,26].

Currently, loess improvement mainly involves the use of lime, fly ash, lignin fiber alone or a combination of both. Osula et al. [27] conducted an investigation into the impact of curing time on loess improvement using lime and cement. Boadman et al. [28] observed that in lime-improved soils, ion exchange and agglomeration of clay particles significantly increase early strength, while volcanic ash reactions primarily enhance later strength. Helenn [29] conducted indoor experiments to investigate the engineering properties of lime-amended soils. Liu and Zhao et al. [30,31] delved into the key factors influencing the compaction, compression, and strength properties of lime-amended loess within the context of the Zhengzhou West High-Speed Railway. Furthermore, Liu [32] and Zhang et al. [33] carried out a series of investigations on lignin-amended loess, revealing that lignin effectively enhances the strength of loess. At the microscopic level, lignin acts to cement soil particles and fill pores. Results from Liu et al. [34] indicated that lignin incorporation effectively boosts the strength of loess while improving its water-holding capacity and water stability. Moreover, Hou et al. [35] delved into a multidimensional analysis of lignin-amended loess, pinpointing calcium lignosulfonate as a novel curing material capable of enhancing particle cementation and structural compaction. Gao [36]and Zhong et al. [37] conducted research on lignin fiber-amended loess using sampling methods, which revealed significant impacts on the strength of ameliorated loess. Varsha studied the use of granite sand (GS) and calcium lignosulfonate (CLS) as sustainable stabilizers that can be mixed with clay. The dosage of GS was 30%, 40% and 50% respectively, and the dosage of CLS was 0.25%, 0.5%, 1% and 1.5% respectively. Direct shear and consolidation tests were carried out on GS-CLS mixed soil samples solidified for 7 days and 14 days. The improved stabilizer improved the shear parameters and consolidation characteristics at the optimum dosage of 30% GS and 0.5% CLS, and the cohesion and internal friction angle increased by 84% and 163%, respectively [38].

Existing studies have mainly focused on the improvement of loess properties by lime or lignin as a single inorganic or organic modifier. The combination of inorganic and organic materials to improve loess is less studied. In addition, lime is a widely distributed, inexpensive and easy to obtain geotechnical engineering materials, will be a certain amount of lime and loess mixed to improve the engineering properties of loess and the formation of lime stabilized loess has been a common method of improving loess and its improved mixing ratios and technology has been relatively mature, but the lime-improved soil is easy to be softened in contact with water, and a large amount of dust will be generated during the construction process. As a kind of organic material with large storage capacity and environmental protection, the application of lignin in loess improvement can greatly improve the engineering performance of loess such as water loss disintegration, softening, etc., and the biological curing agent has the advantages of less pollution and less dust. Therefore, this time, lime and lignin are used to improve loess roadbed together.

Currently, numerical simulation methods for performing roadbed settlement calculations are divided into three main categories: finite element method, finite difference method, boundary element method, finite difference method and boundary element method.The finite element method is widely used in geotechnical engineering calculations because of its simple concept, easy to grasp, accurate results and other advantages.Li Junhong [39] used ABAQUS to simulate the effect of the depth of influence of drainage strips on the consolidation of soft foundations; Alielahi et al. [40] used Settle 3D software to simulate the effect of drainage strips with stacking pre-compression technology to improve the performance of foundations; Kazem et al. [41] pointed out that if the parameters of the soil in the software of Settle 3D were chosen appropriately, it was possible to reasonably estimate the time of settling and consolidation.Luo [42] Simulation of vertical displacement of composite-amended loess roadbed along depth direction using MIDAS/GTS software.Auersch et al [43] established a numerical model of railroad track-roadbed and investigated the distribution law of the dynamic response inside the model of railroad track-roadbed. The distribution of dynamic response inside the track-roadbed model was investigated.

This time, MIDAS/GTS is adopted, which is a finite element analysis and design software developed for geotechnical and tunneling engineering, and it is a special software for geotechnical engineering, applicable to geotechnical, underground structures, tunnels, subways and other fields, and it is able to carry out the analysis of the construction stage, the analysis of the load bearing capacity and deformation, and the analysis of the stability of slopes, and so on. High-speed railroad operation often involves vibration loads, the vibration load research of railroad roadbed is less and vibration load is the focus of railroad roadbed research, MIDAS/GTS provides the corresponding time course analysis method, which can better respond to the settlement of the roadbed in the process of high-speed railway rapid operation.

The Ili region possesses abundant loess deposits, though the loess itself exhibits poor inherent properties. This study will employ a combined organic-inorganic modification approach, utilising the region’s plentiful loess as subgrade material. A novel, low-cost, environmentally friendly, and high-performance ‘lime-lignin synergistic modification’ technology is proposed. The synergistic cementation and stabilisation mechanism of organic (lignin) and inorganic (lime) materials in loess modification will be elucidated. This approach achieves the resourceful, high-value utilisation of industrial solid waste (lignin), delivering significant economic, environmental, and social benefits. Hence, this paper investigates the impact of lime and lignin dosages on the behavior of loess through a comprehensive series of tests including compaction tests, unconfined compression tests, and triaxial shear tests. Additionally, numerical simulations were employed to analyze the effects of lime and lignin at varying dosages on the enhancement of loess for roadbed applications, resulting in the optimal dosage for enhancing loess, as obtained from this study. The findings of this research provide new insights and experimental references for enhancing roadbeds in loess regions.

## 2. Materials and methods

### 2.1. Materials

**Fig 1:**
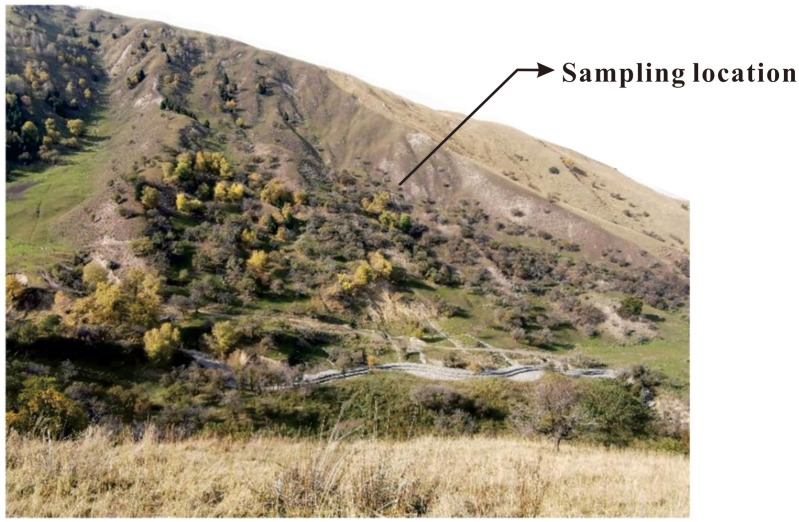
Sampling location map.

**Fig 2:**
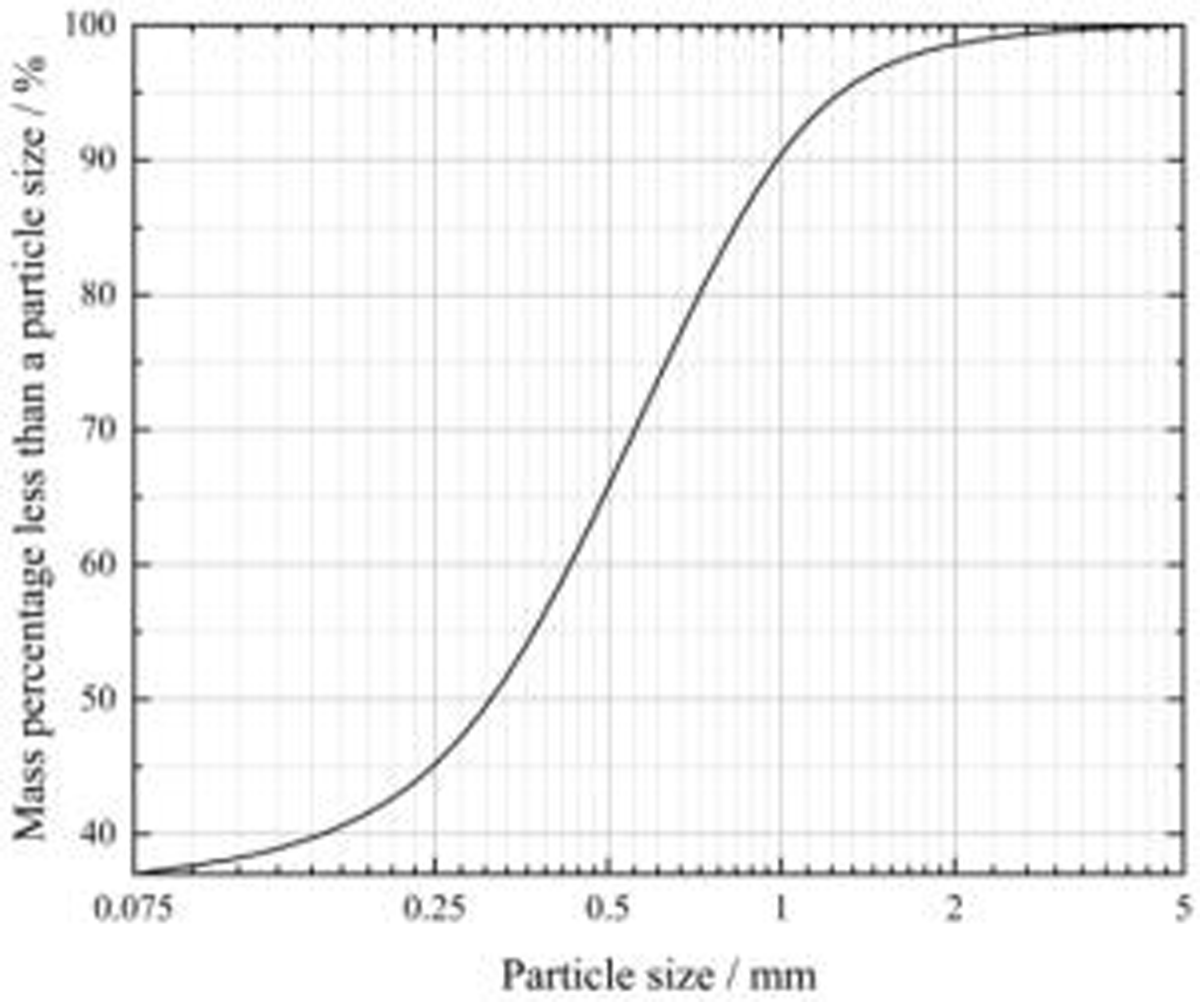
Cumulative curve of particle gradation of Ili loess.

To eliminate the side-effect caused by surface-decaying plants or other disturbances, the loess used in this study was sourced at a depth of 2 meters below the sampling site in Xinyuan County, Ili region, Xinjiang(Fig 1). The loess in the Ili region is characterized by a light yellow colour and devoid of plant roots. Particle size analysis on the tested soil was conducted using the Microtrac Laser Particle Sizing Analyzer, with the results depicted in Fig 2. Basic physical property parameters were measured and are presented in Table 1. According to the GB/T 50123−2019 [44], this kind of loess was classified as low liquid-limit clay. Quantitative X-ray diffraction (XRD) analysis was applied to determine the main mineral composition of the test loam, the results of which is detailed in Table 2. The primary constituents of the test loam were identified as quartz and calcite, among others.

**Table 1:** Basic physical indicators.

Natural moisture content	Natural density	Natural dry density	Saturated moisture content	Plastic limit WP (%)	Liquid limit WL (%)	Porosity ratio	Porosity
20.89%	1.96g/cm^3^	1.64g/cm^3^	24.57%	17.34	27.09	28.34	22.08%

**Table 2:** Mineral composition analysis results.

Quartz	Calcite	Dolomite	Clinochlore	Albite	Muscovite mica	Rutile
28.1%	21.1%	3.2%	10.5%	19.5%	15%	1.5%

The lime used for soil improvement was calcium quicklime powder. The particular lime is readily available in the market, cost-effective, and commonly used in engineering applications. The chemical composition of the lime is listed in Table 3

**Table 3:** Main chemical composition of lime.

Ingredient	CaO	MgO	SiO_2_	Fe_2_O_3_	Al_2_O_3_
Lime	90.47%	2.61%	4.77%	0.37%	0.74%

**Fig 3:**
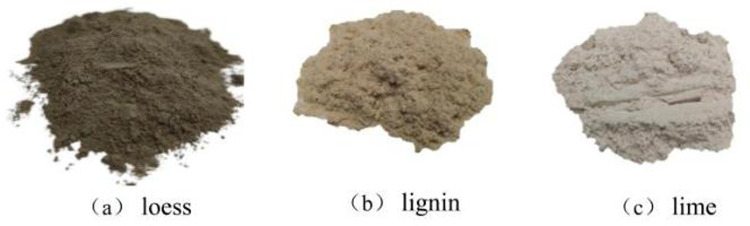
Digital image.

The lignin utilized in the experimental improvement process exhibits a complex chemical structure characterized by its status as a high-polymer organic compound composed of carbon (C), hydrogen (H), and oxygen (O) elements [45]. This organic fiber is derived from the chemical treatment of natural wood and presents itself as a light yellow flocculent solid. The lignin fiber has an approximate length of 1 mm with an average diameter of about 40 µm. It boasts a low water content of less than 5% and an ash content of 18%. Furthermore, it demonstrates heat resistance up to 230 °C, maintains a pH level of 7.0, and has an aromatic odor. Additionally, it possesses excellent qualities, including high toughness, dispersion capabilities, chemical stability, crack resistance, as well as resistance to both acid and alkali corrosion.Photographs of the loess, lime, and lignin materials used in this experiment can be seen in Fig 3

### 2.2. Sample preparation

**Fig 4:**
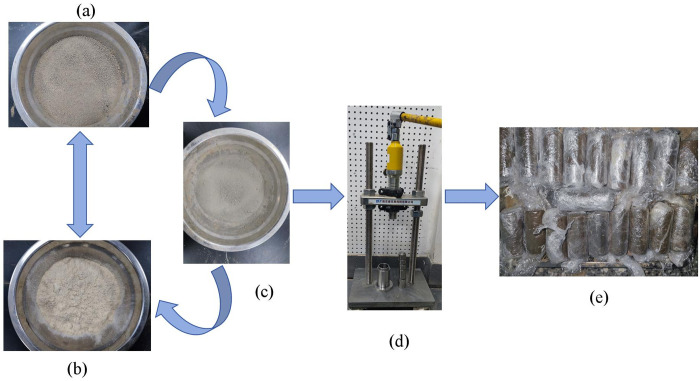
Sample preparation process. (a) Loess; (b) Lignin; (c) Lime; (d) Sampler; (e) Sample.

The loess was initially placing into a drying oven at 105°C for a period exceeding 24 hours. This process continued until the mass of the loess remained constant, signifying that it had reached a dry state. Subsequently, the loess was cooled to room temperature and subjected to screening using a sieve with a mesh size of 2 mm. Simultaneously, the lignin fibers were sieved through a 1 mm mesh sieve to remove any flocculent agglomerates [46]. The subsequent step involved the preparation of a mixture comprising lime, lignin, loess, and water, as specified in Table 4. This mixture underwent thorough mixing and stirring for a duration of 30 minutes, ensuring the consistent integration of soil samples with lignin fibers and lime during the preliminary pre-testing phase. During the pre-laboratory, it was observed that agglomeration occurred within the mixture, involving lignin fiber, lime, and loess. Subsequently, the mixed soil samples were sieved again to mix the lignin fibers, lime and loess well, thus maintaining the homogeneity of the samples. The treated soil samples were sealed for 24h and then made by loading in layers using a hydrostatic loading device. Finally, the prepared specimens were sealed with plastic film and left to stand for 24 hours to allow for the equilibrium of moisture migration within the specimens to begin the test. This study did not consider curing time, focusing solely on the effect of lime and lignin ratios on loess improvement. Therefore, curing time was uniformly set at 24 hours. The specific sample making process for this test can be seen in Fig 4. Soil materials employed in the compaction test were prepared according to the specifications outlined in Table 4. Subsequently, compaction experiments were conducted using the JDS-2 lightweight compaction apparatus in accordance with the GB/T 2019−25 [44]. The objective was to determine the maximum dry density and optimum moisture content for lignin-lime improved loess at varying dosages.

**Table 4:** Compaction test programme.

Serial number	Lime and lignin dosages	Different moisture content gradients	Number of parallel test groups
1	0	15%、17%、19%、21%、23%	3
2	2%:1%	15%、17%、19%、21%、23%	3
3	4%:2%	15%、17%、19%、21%、23%	3
4	6%:3%	15%、17%、19%、21%、23%	3
5	8%:4%	15%、17%、19%、21%、23%	3

### 2.3. Unconfined compressive strenght test

**Fig 5:**
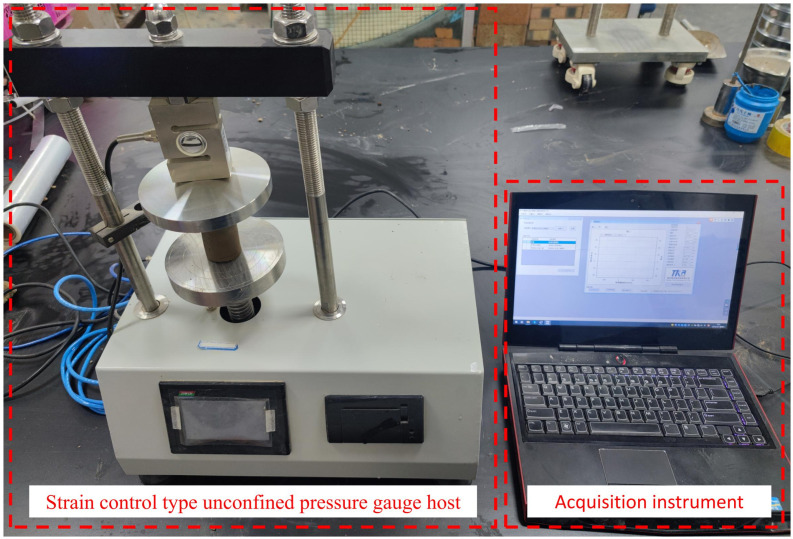
Unconfined compression test instrument.

Unconfined compression strength serves as a key indicator for evaluating the effectiveness of improved materials in soil treatment. The unconfined compressive strength test (UCS) results were utilized to evaluate the curing effect of lignin-lime on loess and to determine the optimal dosage. According to the existing studies on the strength of lime stabilized soils at different lime contents, the optimum lime content is about 5% to 9% [47,48] and the optimum lignin content available to improve the strength of foundation soils have been determined by unconfined compressive tests and triaxial tests. They showed that the strength of modified soil was maximum when the lignin content was between 2% and 12% [49–51], through the previous studies, then pre-tests of dosing were carried out prior to the commencement of the test found that the soil clumped severely when the lignin dosing was too high above 5%, therefore, according to the lime and lignin 0%, 2%:1%, 4%:2%, 6%:3%, and 8%:4% dosing to prepare 39.1 mm x 80 mm cylindrical soil samples.These samples were subsequently subjected to uniaxial compressive strength testing using an STK. WCX-II unconfined compressive strength tester (Fig 5). The testing protocol involved applying a loading rate of 2% per minute while closely monitoring the real-time test curves. The test was halted once the specimen’s strain value had further changed by 3% to 5% after the curve reached its peak.

### 2.4. Triaxial shear test

**Fig 6:**
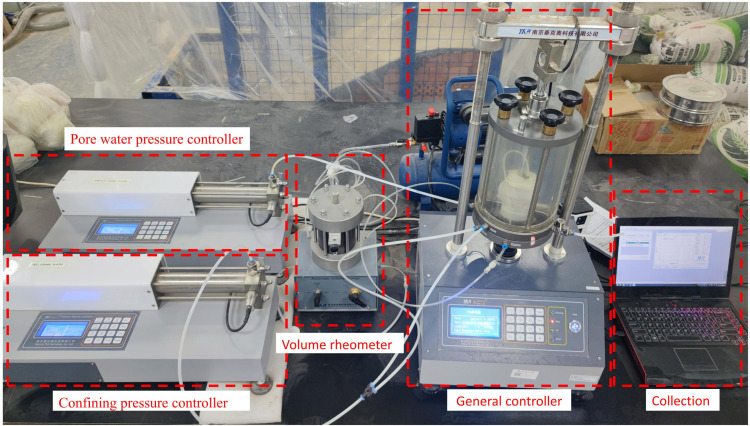
Triaxial testing instrument.

Given that no sufficient time are allowed for drainage consolidation of the loess roadbeds which is subjected to transient vehicle loads, this study investigates the shear properties through the unconsolidated-undrained (UU) test. The TFB-1 type unsaturated soil stress-strain control triaxial apparatus was adopted (Fig 6). Following the GB/T50123-2019 [44], cylindrical soil samples of dimensions 39.1 mm × 80 mm were prepared for different lime and lignin mixing dosages: 0%, 2%: 1%, 4%: 2%, 6%: 3%, and 8%: 4%. A shear strain rate of 0.5 mm/min (0.5%/min to 1.0%/min) was applied, and the control strain was set at 20% of the axial strain. The damage point on the stress-strain curve was defined as the peak value of the principal stress difference when such a peak existed. If no peak value was observed, the damage point was determined when the axial strain reached 15%. The confining pressures were set at 100kPa, 200kPa, and 300kPa, respectively.

## 3. Results

### 3.1. Analysis of compaction test results

**Fig 7:**
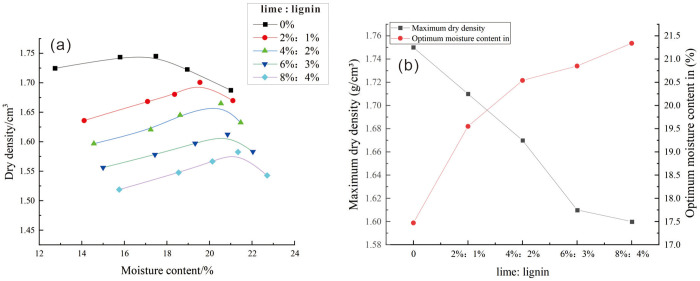
Test results of compaction characteristics. (a) Compaction curves; (b) Optimum moisture content and maximum dry density.

The results of the compaction test for lignin-lime improved loess are presented in Fig 7 and Table 5. It can be seen from Fig 7 (a) that the maximum dry density of the amended loess gradually decreases with increasing proportions of lignin fiber and lime, while the optimum moisture content exhibits an ascending trend in comparison to plain soil. The optimum moisture content increased by 3.87%, and the maximum dry density decreased by 0.15 g/cm^3^ for the highest dosage considered. The rise in optimum moisture content can be attributed to the chemical reaction between lime and water, which consumes a portion of the water. Additionally, lignin fiber is known to absorb water, which further contributes to the higher optimum moisture content as the dosages increase. The gradual reduction in maximum dry density can be attributed to several factors. Firstly, both lime and lignin have lower specific gravity compared to loess, which inherently lowers the overall density of the mixture. Additionally, the addition of lime and lignin induces a chemical reaction with soil particles, resulting in the formation of cementitious bonds. This causes the finer soil particles to coalesce into clusters, altering the structural properties of the soil and enhancing its internal cohesion. Consequently, the maximum dry density of the modified soil is lower when compared to the native loess.

**Table 5:** Maximum dry density and maximum moisture content of modified soils.

Modifier dosage (lime: lignin)	0	2%:1%	4%:2%	6%:3%	8%:4%
Optimum moisture content in %	17.47	19.55	20.54	20.85	21.34
Maximum dry density g/cm³	1.75	1.71	1.67	1.61	1.60

Fig 7(b) illustrates a noteworthy trend: the maximum dry density experiences a more rapid decline during the initial stage, followed by a gentler reduction in the later stage. Simultaneously, the optimum water content demonstrates a swifter increase initially, followed by a more gradual rise in the later stage. This phenomenon can be attributed to soil internal reactions. In the early stages, there is a rapid chemical reaction between lime and water, which quickly consumes a significant amount of water [52]. Lignin fibers become encapsulated, leading to the formation of a stable agglomeration structure. This structural change reduces the pore space within the soil and enhances soil cohesion. In addition, pore water between soil particles is consumed by lime and lignin. Consequently, the water film surrounding the particle surfaces becomes thinner, necessitating inter-particle movement to overcome greater resistance and expend more energy. This results in a rapid initial increase in optimum moisture content and a correspondingly rapid decrease in maximum dry density. At a later stage, as the lime and lignin content increases, they facilitate the formation of an agglomerated structure with ease. This structure results in the creation of a hollow system within the soil skeleton, subsequently reducing cohesion. Compared to the previous stages, where particles had to move against each other to overcome greater resistance, the consumption of energy is lessened. Consequently, the rate of reduction in maximum dry density decelerates. Additionally, due to the influence of the agglomerated structure, the contact between lignin, lime, and water is reduced, further contributing to a slow increase in optimum moisture content.

In summary, lime and lignin not only fill the large pores between loess particles, but also absorb large amounts of water and promote the production of more hydrates, making the soil structure more stable.

### 3.2. Analysis of the results of the unconfined compressive strength test

**Fig 8:**
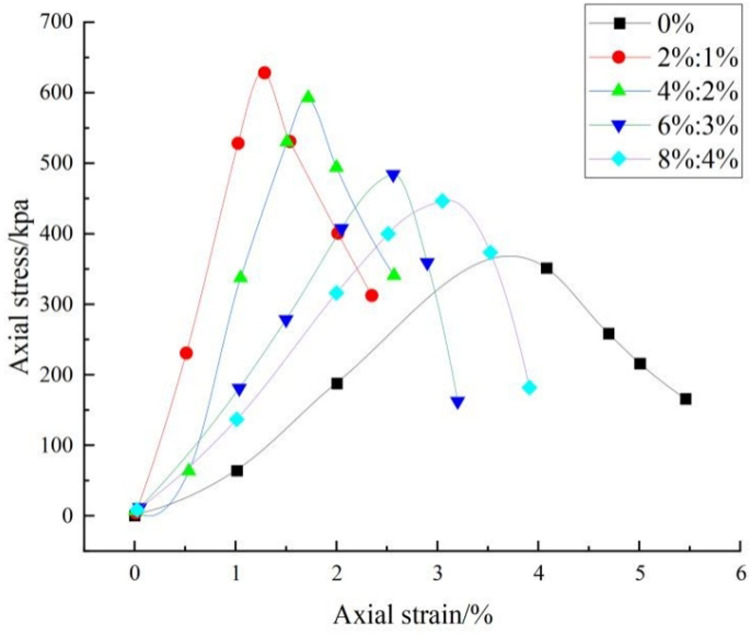
Stress-strain curve of modified loess.

The stress-strain curves of the modified loess obtained from the unconfined compressive strength test are shown in Fig 8. It is in Fig 8 that the stress-strain relationship of the improved loess exhibits a trend similar to that of the stress-strain curve of the untreated soil.However, it’s noteworthy that the peak axial stresses observed in the improved soil specimens, with varying lime and lignin dosages, exceed those of the untreated soil. This observation suggests that the addition of lime and lignin into loess effectively enhances the axial stress capacity of the soil. Furthermore, the peak axial stresses of the improved loess exhibit an initial increase followed by a subsequent decrease as the lime and lignin content in the soil increases. The maximum axial stress in the improved loess is achieved with a lime-to-lignin dosage of 2%:1%, the compressive strength of loess increased by 73.97%.

As illustrated in Fig 8, the stress-strain curve experiences a rapid decline after reaching its peak upon the addition of lime and lignin, suggesting increased soil brittleness due to these additives. For samples treated with various lime and lignin contents, the initial stress-strain curves exhibit steepness, with stress increasing significantly faster than strain. The incorporation of lime and lignin initially strengthens the loess, increasing its resistance to cracking and strengthening the soil structure. Fig 8 also demonstrates that as lime and lignin content increases, the reduction rate of the stress-strain curve peak gradually diminishes at a slower speed after reaching its peak value. In the stress-strain curve, the steeper the curve, the greater the stiffness of the material. The slope of the curve reflects the elastic modulus of the material. The greater the slope, the greater the stress required by the material under the unit strain, that is, the greater the ability of the material to resist deformation, and the greater the stiffness [53]. This phenomenon indicates that lignin addition mitigates the brittle failure of the soil. This effect can be attributed to lignin’s ability to facilitate the formation of an internal network structure within the soil matrix. This network connects soil particles, encapsulating them and enhancing the soil’s deformation resistance. Consequently, the soil’s failure exhibits a tendency to shift from brittle failure to plastic failure.

### 3.3. Analysis of triaxial shear test results

**Fig 9:**
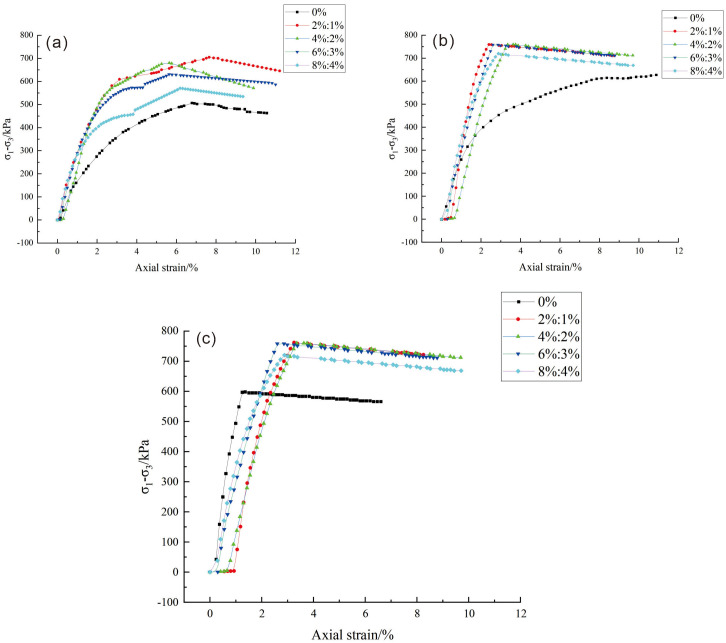
Stress-strain curve. (a) 100kPa; (b) 200kPa; (c) 300kPa.

The stress-strain curves of loess amended with different dosages of lime and lignin fibers are shown in Fig 9. The amended loess exhibits strain-hardening behavior when subjected to confining pressures of 100 kPa and 200 kPa. However, when subjected to a confining pressure of 300 kPa, the stress-strain curve shifted from strain hardening to strain softening. This shift is attributed to the effect of increasing perimeter pressure on the soil stress-strain behavior, highlighting the important effect of the magnitude of the perimeter pressure on the soil response. By contrast, the improved soil exhibits strain-softening behavior in all cases (100 kPa, 200 kPa, and 300 kPa). This distinction is primarily a result of the incorporation of lime and lignin into the soil., with lignin fibers producing matter possessing cementation and curing properties when mixed with water. Consequently, the improved soil displays a certain level of brittleness. This brittleness is further accentuated by the agglomerates formed through the hydration of lime with water, leading to a transition from plastic to brittle failure, which results in strain-softening behavior in the soil.

It is also evident in Fig 9 that the principal stress difference typically reaches its peak during the shear process of the specimens under varying confining pressures. Moreover, compared to remodeled loess specimens lacking lime and lignin, those with a certain quantity of lime and lignin exhibited an enhancement in their principal stress difference to some extent. A closer examination of the stress-strain curves under different confining pressures reveals that an increase in confining pressures corresponds to an augmentation in the principal stress difference of the specimens. Specifically, the principal stress difference is greatest under a confining pressure of 300 kPa, followed by 200 kPa as the second highest and 100 kPa as the lowest. This trend arises from the increase in shear strength of the improved loess, which can be attributed to the increasing pressure progressively compressing the volume of the improved loess specimen under the same lime and lignin fiber content. As a result, the soil particles move closer together, increasing the interparticle interaction forces, increasing the cohesive forces between the soil particles, and increasing the normal stress along the relative slip surface (i.e., the shear surface). This diminishes weak structural planes and fissures during the shear process, along with the gradual decrease of the shear deformation phenomenon of the specimen. The compaction effect of the perimeter pressure concurrently bolsters the soil’s resistance to deformation.

As highlighted in Fig 9, the lime-to-lignin dosage of 2%:1% exhibits the highest curve position, signifying the most effective improvement. When the content of lime and lignin exceeds 2%: 1%, the stress-strain curve of the sample shows a sharp and brittle strength loss after reaching the peak strength. This is because the synergistic effect of lime and lignin makes the loess sample change from plastic failure to brittle failure.

**Fig 10:**
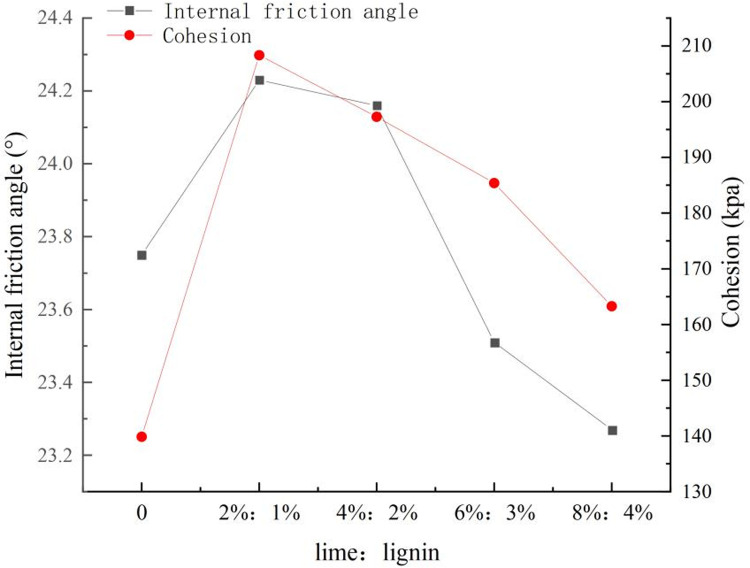
Variation curves of cohesion and angle of internal friction.

Fig 10 provides additional insight into the shear strength parameters of lime-lignin amended loess. The friction angle and cohesion of soil samples were calculated by Mohr-Coulomb method, according to the test data, the Mohr circle is drawn to obtain the strength envelope of the soil. The inclination angle of the strength envelope is the friction angle of the soil sample, and the intercept with the longitudinal axis is the cohesion. As the lime content increases, the cohesion parameter (c) and the internal friction angle (φ) of the improved loess exhibit an initial increase followed by a subsequent decrease. The parameters cohesion (c) and angle of internal friction (φ) reached their maximum values at 2%:1% lime and lignin dosing, with maximum values of 208.35 kPa and 24.23°, which were about 149% higher than that of the natural loess.

## 4. Numerical simulation

### 4.1. Model introduction

**Fig 11:**
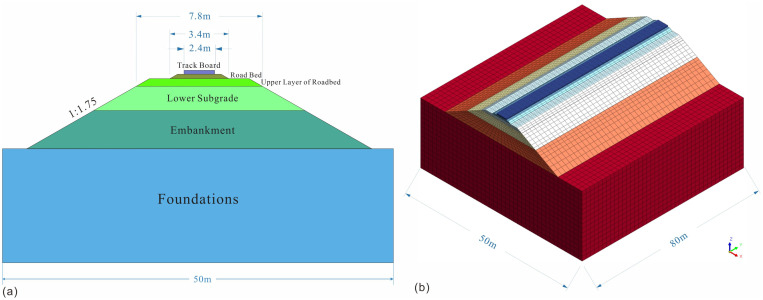
Railway roadbed model.

In compliance with the TB10001−2016 [54], a finite element model for the roadbed of the passenger and freight dual-use electrified railway train was constructed using MIDAS GTS-NX software. The high-speed railway roadbed model is discretized into 55,218 cells and comprises the following key components: track board, ballast bed, upper roadbed layer (comprising graded gravel), lower roadbed layer (representing the improved section), embankment (constructed of loess), and foundation (comprised of loess). The model features a cross-sectional length and height of 50 meters and 21.15 meters, respectively, as depicted in Fig 11(a). The longitudinal length of the roadbed spans 80 meters, as illustrated in Fig 11(b). Additionally, the sub-base layer of the high-speed railway possesses a thickness of 1.9 meters, making it a crucial part of the roadbed improvement efforts. The parameters for cohesion and internal friction angle characterizing both loess and improved loess were determined through triaxial shear tests, while the elastic modulus was derived from unconfined compressive strength tests. Detailed computational parameters for the finite element model can be found in Table 6.

**Table 6:** b Calculation parameters of the finite element model.

Placement	Filling material	Thicknesses/m	Elastic modulus/MPa	Poisson’s ratio	Density kg/m3	Angle of internal friction/kpa	Cohesion (°)
Track board	/	0.3	36000	0.2	26000	/	/
Ballast bed	/	0.35	220	0.25	2100	/	/
Subgrade surface	Graded gravel	0.6	200	0.3	2100	18	30
Sub-base layer of subgrade	Loess	1.9	65	0.32	1750	23.75	139.8426
	Lime: lignin 2:1		528	0.27	1710	24.23	208.3513
	Lime: lignin 4:2		337	0.29	1670	24.16	197.2821
	Lime: lignin 6:3		180	0.3	1610	23.51	185.3877
	Lime: lignin 8:4		136	0.3	1600	23.27	163.2797
Embankment	Loess	3	65	0.32	1750	23.75	139.8426
Foundation	Loess	15	45	0.32	1750	20.63	109.2345

### 4.2. Numerical simulation results

**Fig 12:**
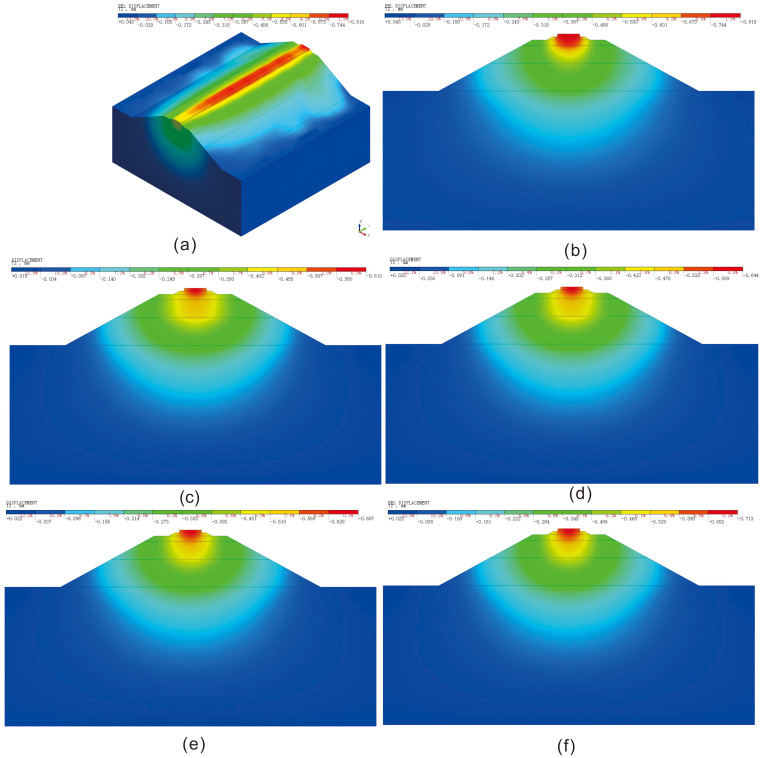
Vertical displacement cloud diagram of lime-lignin improved railway roadbed. (a) 0%; (b) 0%; (c) 2%:1%; (d) 4%:2%; (e) 6%:3%; (f) 6%:3%.

**Fig 13:**
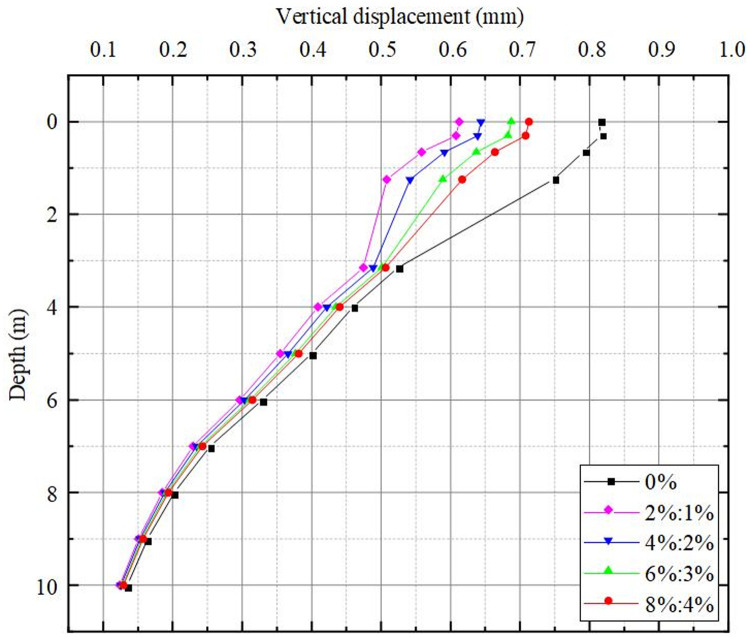
Vertical displacements at different depths of lime-lignin improved railway roadbed.

The vertical displacement cloud diagram of the roadbed improved with varying lime and lignin dosages is presented in Fig 12, while Fig 13 provides the statistical graph depicting the vertical settlement at various depths of the roadbed undergone different lime and lignin treatments. The statistical depths considered are 0 m, 0.3 m (ballast bed surface), 0.65 m (subgrade surface layer), 1.25 m (surface of the sub-base layer of subgrade), 3.15 m (embankment surface), 4 m, 5 m, 6 m, 7 m, 8 m, and 9 m, with a final depth of 10m. During the passage of a single train, noticeable settlement occurs in the unimproved railway roadbed, with the maximum settlement reaching approximately 0.816 mm.

It is evident from Fig 12-Fig 13 that the vertical displacement distribution pattern in loess roadbeds, which were improved with varying lime and lignin dosages, remains consistent as the depth of the roadbed increases. As shown in Table 7, the deformation within the sub-base layer of loess roadbed improved with different dosages of lime and lignin (that is,0%, 2%:1%, 4%:2%, 6%:3%, 8%:4%)are 0.224 mm, 0.0332 mm, 0.0533 mm, 0.0881 mm, 0.1105 mm respectively. The results indicate that the modified soil effectively reduces vertical settlement displacement in the bottom layer of subgrade. When the dosage of lime and lignin is 2%:1%, the settlement in the bottom layer of the subgrade is the smallest, indicating the most favorable improvement effect. This is attributed to the combination of lime and lignin with water to form polymers, and the lignin also forms a three-dimensional lattice in the soil, which strengthens the interconnections between the polymers and enhances the bonding between the fillers. As a result, the pore ratio in the soil is reduced, leading to an increase in compression modulus and a corresponding reduction in deformation.

**Table 7:** Subgrade bed displacement of improved railway subgrade.

Lime: lignin	0%	2%:1%	4%:2%	6%:3%	8%:4%
Surface stress of the upper layer of subgrade/mm	0.791	0.5582	0.5913	0.6371	0.6641
Surface stress of the bottom layer of subgrade/mm	0.748	0.5079	0.542	0.589	0.6167
Stress at the base of the bottom layer of subgrade/mm	0.524	0.4747	0.4887	0.5009	0.5062

**Fig 14:**
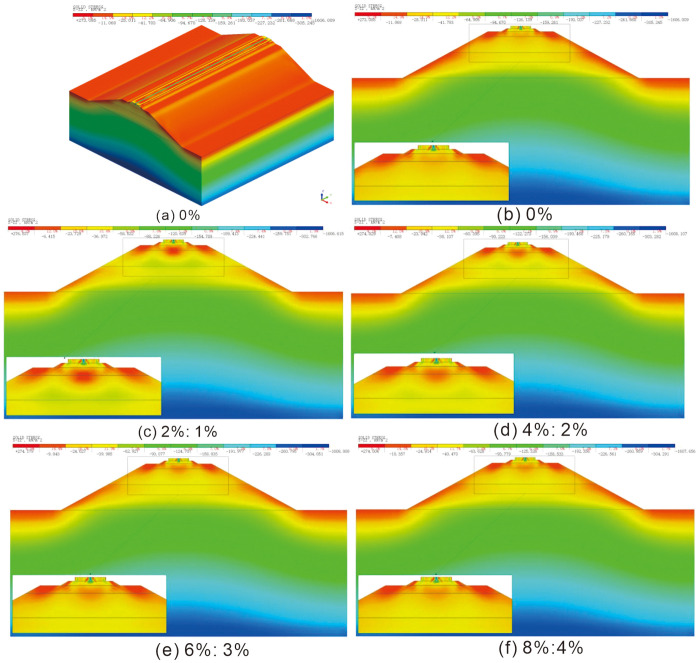
Stress cloud diagram of lime-lignin improved railway roadbed. (a) 0%; (b) 0%; (c) 2%:1%; (d) 4%:2%; (e) 6%:3%; (f) 8%:4%.

**Fig 15:**
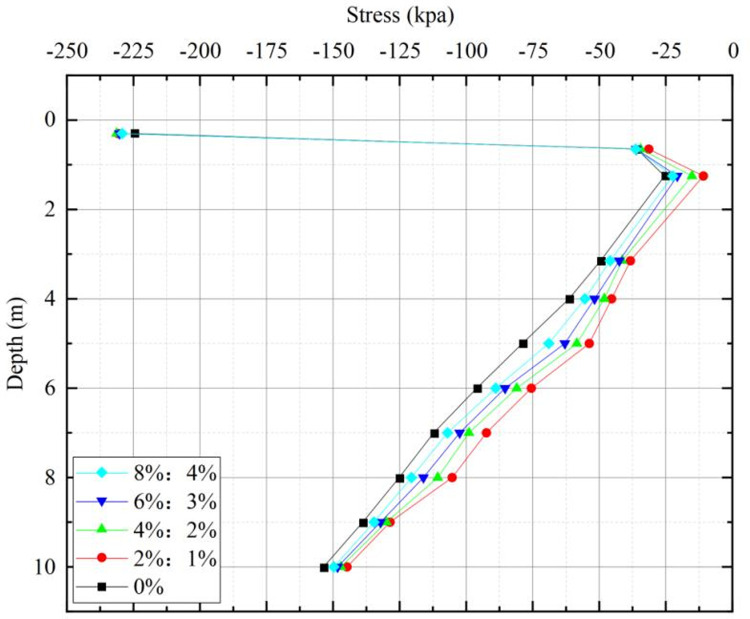
Stresses at different depths in lime-lignin improved railway roadbed.

The stress cloud diagram for roadbeds improved with varying lime and lignin dosages is depicted in Fig 14, while Fig 15 presents the statistical distribution of stress at different depths of roadbeds subjected to these dosage variations. The statistical depths correspond to those used in the vertical settlement analysis.

A similar pattern in the stress distribution for improved loess under different lime and lignin dosages, namely 0%, 2%:1%, 4%:2%, 6%:3%, and 8%:4%, as illustrated in Fig 15. The result shows that the stress initially decreases and then increases as the depth of the roadbed increases. At a depth of 1.25 m, the stress trend shifts from decreasing to increasing. This shift occurs because the self-weight stress continues to rise as the depth increases. Simultaneously, the static load pressure from the upper train load spreads and decreases with increasing depth. There is a turning point where stress transitions from decreasing to increasing at the depth of 1.25 m. This transition is primarily due to the fact that the static load of the top train has minimal effect on the stress at greater depths, and the stress is primarily influenced by the self-weight of the upper soil layers, which increases with depth.

Table 8 presents data on stress attenuation amplitude within the sub-base layer of subgrade when different dosages of lime and lignin-amended loess are used. This attenuation decreases from 27.44 kPa at 2%:1% to 24.05 kPa at 0%. At the same time, the stress at the bottom position of the sub-base layer of subgrade increases from 38.30 kPa to 49.55 kPa. These findings indicate that lime-lignin amended loess fillers can effectively and rapidly diffuse the stress from upper load. This effect is due to the lime lignin modified loess, lignin fibers connect the agglomerates and particles in the soil, the stress can be effectively transferred to all parts of the soil through the lignin fibers, allowing the stress to diffuse quickly thus making its overall modulus of elasticity higher than that of the plain soil. As the thickness of the improved loess increases, the cementitious material present in the soil enhances the integrity of the roadbed filler. This results in the upper static load spreading along the direction of travel, reducing the downward transfer of load and subsequently decreasing stress on the roadbed below the bottom layer of the subgrade. As a result, settlement is reduced, contributing to the overall stability improvement of the railway roadbed.

**Table 8:** Improved railway roadbed subgrade stress.

Lime: Lignin	0%	2%:1%	4%:2%	6%:3%	8%:4%
Surface stress of the upper layer of subgrade/kPa	−35.85	−31.40	−34.50	−36.08	−36.31
Surface stress of the bottom layer of subgrade/kPa	−25.50	−10.86	−15.17	−20.68	−22.41
Stress at the base of the bottom layer of subgrade/kPa	−49.55	−38.31	−41.44	−42.47	−45.95

**Fig 16:**
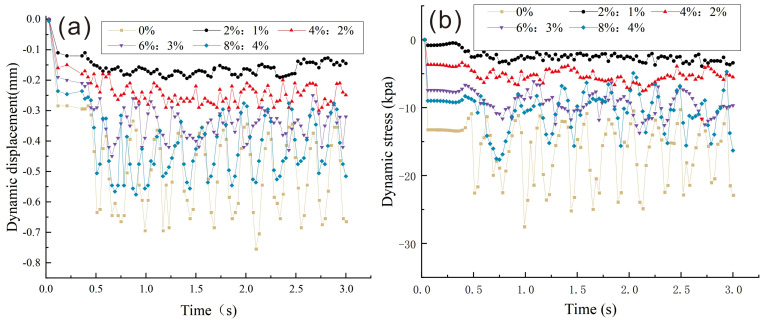
Dynamic displacement and dynamic stress statistics under dynamic loading condition. (a) Time-course curves of dynamic displacements on the surface of the upper layer of subgrade; (b) Time-course curve of dynamic stress on the surface of the upper layer of subgrade.

In Fig 16(a), the time course curve of displacement of the upper layer of the subgrade exhibits significant variations in vertical displacement with different dosages of lime-lignin amended loess. The vertical displacement fluctuation is notably smaller when a lime and lignin dosage of 2%:1% is used in the sub-base layer of subsurface. By contrast, the time course curve of the plain loess exhibits pronounced fluctuations when lime and lignin are not added. This behavior is primarily related to the strength of the pavement. During high-speed train operations, a substantial dynamic response occurs, leading to greater vertical deformation of the roadbed when weaker fillers are used. Conversely, when the thickness of the improved soil filling is relatively large, the cohesive product generated from lime-lignin amended loess in the appropriate proportion exhibits higher strength and plateticity, resulting in greater resistance to the impact of dynamic train loads. Therefore, when a lime and lignin mixture of 2%:1% is used, train travel is relatively smooth.

In Fig 16(b), the dynamic stress distribution curve of the surface of the upper layer of subgrade reveals significant variations in stress fluctuations with different dosages of lime-lignin amended loess. When a lime and lignin dosage of 2%:1% is used in the sub-base layer of the subgrade, dynamic stress fluctuations are small. Conversely, the time course curve of the plain soil without lime and lignin shows significant fluctuations. This indicates that the cementitious substance present in the improved loess soil, with an appropriate dosage of lime and lignin, enhances the integrity of the roadbed fill. In addition, it effectively and rapidly dissipates the dynamic stress transmitted from the upper load. This phenomenon can be attributed to the improved loess with higher compressive strength, shear strength, and deformation resistance compared to plain loess.

## 5. Discussion

### 5.1. Mechanism of improvement

**Fig 17:**
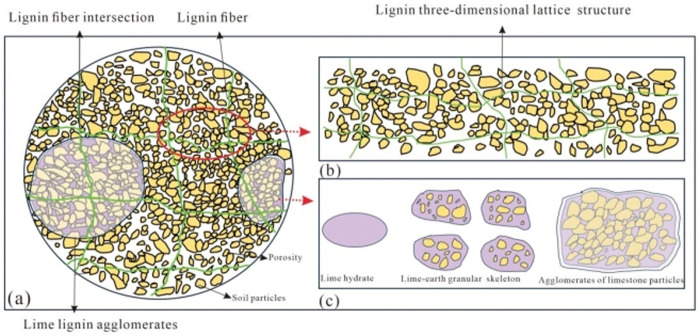
Schematic diagram of the micro-mechanism of lime-lignin amended loess.

The unconfined compressive strength test and triaxial shear test revealed a significant improvement in the angle of internal friction, cohesion, and compressive strength of the lime-lignin improved loess. Lime and lignin played critical but different roles in this improvement, as depicted in Fig 17.

Loess primarily consists of chemical components such as SiO2、Al2O3 and CaO. When exposed to humid conditions, an alkaline component,Ca(OH)2, is generated due to the interaction of water in loess with CaO found in lime. Ca(OH)_2_ undergoes both physicochemical and chemical reactions with the primary chemical constituents of loess, resulting in the formation of compounds such as cementing calcium silicate compounds (mCaO·SiO_2_·nH_2_O), calcium aluminate hydrate (mCaO·Al_2_O_3_·nH_2_O), and Calcium-aluminum feldspar hydrate (mCaO·Al_2_O_3_·SiO_2_·nH_2_O) [55,56]. These compounds create a cement structure within the soil, forming a cement-soil skeleton that prevents particles from easily sliding relative to each other and thus increasing inter-particle friction. With increasing lime content, the volume of these hydrates expands, producing a gel that envelops soil particles to form the skeleton, further strengthening the structural strength of the soil.

The inclusion of lignin significantly enhances both the compressive and shear strength of the soil which can be explained by several mechanisms. Firstly, when lignin fibers mix with water during the sampling process, they produce part of the fluid that acts as a cementing agent, thereby strengthening the bond between soil particles. Additionally, the addition of lignin leads to the formation of a three-dimensional grid structure, which acts as a bridge within the soil to further enhance particle cohesion. This grid structure, formed by lignin, can effectively withstand the tensile stresses induced by axial loads. Furthermore, lignin fibers enhance the soil’s internal stress transfer capacity, reducing stress concentration. They also restrict lateral deformation to a certain extent and hinder slip between soil particles. The above mechanisms result in an overall improvement in the mechanical properties of improved loess. However, excessive addition of lignin fibers can lead to fiber agglomeration, which disrupts their bonding with soil particles. The contact surface between the particles is reduced, resulting in a weakening of the occlusion between the soil particles within the specimen, which increases the void space between the soil particles in the soil body. In severe cases, this can lead to cracking and the formation of weak surfaces within the soil, making it more susceptible to shear failure.

In summary, a cement-soil skeleton was formed between the lime hydrate and the soil particles. The lignin fibers provided an effective stress transfer mechanism and reduced the stress concentration. Thus, the overall mechanical properties of the specimens were improved, but excessive addition had the opposite result. From the unconfined compressive strength, triaxial shear test and numerical simulation, it can also be found that the proper ratio of lime lignin (2%:1%) can effectively increase the roadbed strength, but when lime and lignin are overloaded instead, the roadbed strength is reduced. This result is consistent with its micro-mechanism. The micro-mechanism is mutually verified with the indoor tests and numerical simulations.

### 5.2. Environmental benefits of improved materials

This study found that when the ratio of lime to lignin was 2: 1, the improved loess obtained the best mechanical properties. The significance of this ratio is that it is combined with lignin and lime to achieve a near or better improvement effect than traditional high-dose lime or low-dose cement. This means that the scheme proposed in this study can significantly reduce the dependence on cement materials under the premise of meeting the same subgrade strength standards. The synergistic effect of lignin and lime is not only reflected in the improvement of mechanical properties, but also in the optimization of environmental benefits. The three-dimensional network structure formed by lignin fibers enhances the integrity of the soil, so that less lime can achieve the design strength. This ‘ small amount of high efficiency ‘ mode itself means lower carbon emissions in the process of material production and transportation [57].

### 5.3. Long-term durability

Dry-wet cycle resistance: The composite structure formed in this study with lime hydrate as skeleton and lignin fiber as reinforcement is expected to effectively inhibit the random development of dry shrinkage cracks. However, repeated drying-wetting cycles may still weaken the fiber-matrix interface at the micro level. Its long-term effects need to be quantitatively evaluated by standard dry-wet cycle tests.

Anti-freeze-thaw cycle ability: Lime improvement itself can reduce the plastic limit and optimal water content of soil, which is helpful to reduce the sensitivity of frost damage. The confinement of lignin fibers may also inhibit the growth of ice crystals. However, the advantages and disadvantages of its freeze-thaw resistance need to be strictly judged by its strength loss rate and mass loss rate through freeze-thaw cycle test.

Biodegradability: This is the core concern of lignin application. It is worth noting that the highly alkaline environment (pH > 10.5) created by lime has a strong inhibitory effect on most microorganisms. Therefore, we speculate that the biodegradation rate of lignin will be extremely slow before the alkaline environment of this system is completely neutralized (such as through long-term carbonation or acid rain erosion). Validation of this hypothesis requires long-term field monitoring or accelerated biodegradation tests.

Lime-lignin improved loess technology shows excellent practical feasibility. In terms of field application, its construction technology is highly compatible with the existing equipment and technical system. It only needs to increase the spraying process of lignin slurry, which is easy to implement. In terms of economy, low-cost lignin industrial waste is used to achieve the goal of ‘ low cost and high performance ‘ through optimized synergistic ratio, and has significant life cycle cost advantages. In terms of environmental sustainability, this technology directly reduces the use of high-carbon materials such as cement and lime by recycling large amounts of solid waste, and has obvious carbon emission reduction benefits. It is a powerful candidate to promote the green transformation of engineering materials.

## 6. Conclusions

The systematic laboratory experiments and numerical simulations were carried out in the present research to investigate the mechanical properties of lime-lignin amended loess subgrade. Based on the laboratory tests and numerical simulations, the following conclusions can be drawn:

(1) With an increase in the proportion of lignin fiber and lime, the maximum dry density of the amended loess gradually decreases, while the optimum moisture content shows an increasing trend.
(2) Both the compressive and shear strengths of the amended loess initially increase and then decrease with higher lime-lignin content. The highest compressive and shear strengths of the amended loess are achieved with a lime-to-lignin dosage of 2% to 1%, indicating the most effective improvement.
(3) Under static or dynamic loading, the settlement and stress transfer of loess improved with different mixing ratios of lime lignin show better results than that of plain soil, with the best improvement effect observed when the mixing ratio of lime lignin is 2% to 1%.

This study systematically reveals the mechanical properties and optimization patterns of lime-wood lignin modified loess, identifying a 2% lime and 1% wood lignin ratio as the optimal configuration. The findings provide critical parameters and theoretical foundations for the application of such materials in roadbed engineering. The research not only validates the effectiveness of lime-wood lignin modification technology in enhancing compressive strength, controlling settlement, and improving stress transfer in roadbeds, but also paves new research pathways for developing and applying eco-friendly soil improvement materials in the future.

## References

[1] Dassekpo J-BM, Zha X, Zhan J. Synthesis reaction and compressive strength behavior of loess-flflyash based geopolymers for the development of sustainable green materials. Constr Build Mater. 2017;141:491–500.

[2] Jia L, L C, Guo J. Mechanical properties of lime-fly ash-sulphate aluminum cement stabilized loess. J Renew Mater. 2020;8:17.

[3] Xu Z, Lin Z, Zhang M. Loess in China and loess landslides. Chin J Rock Mech Eng. 2007;26:1297–132.

[4] Li Y. A review of shear and tensile strengths of the Malan Loess in China. Eng Geol. 2018;236(4):4–10.

[5] Assallay AM, Rogers CDF, Smalley IJ. Formation and collapse of metastable particle packings and open structures in loess deposits. Eng Geol. 1997;48:101–15.

[6] Zuo L, Xu L, Baudet BA, Gao C, Huang C. The structure degradation of a silty loess induced by long-term water seepage. Engineering Geology. 2020;272:105634. doi: 10.1016/j.enggeo.2020.105634

[7] Cao HY, Jia DB, Chen TJ. Study on Deformation Characteristics of Deep Foundation Pit in Unsaturated Soil. AMR. 2011;374–377:1809–12. doi: 10.4028/www.scientific.net/amr.374-377.1809

[8] Qian SZ. Study on the Effect of Water Content on Shearing Strength Parameters of Unsaturated Loess. AMM. 2014;638–640:585–8. doi: 10.4028/www.scientific.net/amm.638-640.585

[9] Wang Y, Xie W, Gao G. Effect of different moisture content and triaxial test methods on shear strength characteristics of loess. E3S Web Conf. 2019;92:07007. doi: 10.1051/e3sconf/20199207007

[10] Yang W, Pan B, Jin L, Wang Y, Saleem F. Experimental study on dynamic characteristics of Qingyang loess under different water contents. Arab J Geosci. 2020;13(19). doi: 10.1007/s12517-020-05989-1

[11] Sheng-you LEI, Zhi YLI, Ji Q, Wang, Zhao LIU. Effect of water content on strength of unsaturated loess. Jiaotong Yunshu Gongcheng Xuebao. 2012;12:1–5. doi: 10.19818/j.cnki.1671-1637.2012.01.001

[12] Leng Y, Peng J, Wang Q, Meng Z, Huang W. A fluidized landslide occurred in the Loess Plateau: A study on loess landslide in South Jingyang tableland. Engineering Geology. 2018;236:129–36. doi: 10.1016/j.enggeo.2017.05.006

[13] Kim Y, Park H, Jeong S. Settlement Behavior of Shallow Foundations in Unsaturated Soils under Rainfall. Sustainability. 2017;9(8):1417. doi: 10.3390/su9081417

[14] Zhou YF, Tham LG, Yan WM, Dai FC, Xu L. Laboratory study on soil behavior in loess slope subjected to infiltration. Eng Geol. 2014;183:31–8. doi: 10.1016/j.enggeo.2014.09.010

[15] Garakani AA, Haeri SM, Khosravi A, Habibagahi G. Hydro-mechanical behavior of undisturbed collapsible loessial soils under different stress state conditions. Eng Geol. 2015;195:28–41. doi: 10.1016/j.enggeo.2015.05.026

[16] Feng SJ, Du FL, Shi ZM, Shui WH, Tan K. Field study on the reinforcement of collapsible loess using dynamic compaction. Eng Geol. 2015;185:105–15.

[17] Zhang YJ, Yu CX, L F, et al. Experimental study on asbestos fiber reinforced fly ash soil-cement for soft soil enhancement. Journal of Engineering Geology. 2015;23(5):982–8.

[18] Ghazavi M, Roustaei M. Freeze–thaw performance of clayey soil reinforced with geotextile layer. Cold Regions Sci Technol. 2013;89:22–9. doi: 10.1016/j.coldregions.2013.01.002

[19] Kravchenko E, Liu J, Niu W, Zhang S. Performance of clay soil reinforced with fibers subjected to freeze-thaw cycles. Cold Regions Science and Technology. 2018;153:18–24. doi: 10.1016/j.coldregions.2018.05.002

[20] Olgun M. The effects and optimization of additives for expansive clays under freeze–thaw conditions. Cold Regions Science and Technology. 2013;93:36–46. doi: 10.1016/j.coldregions.2013.06.001

[21] Eskişar T, Altun S, Kalıpcılar İ. Assessment of strength development and freeze–thaw performance of cement treated clays at different water contents. Cold Regions Sci Technol. 2015;111:50–9. doi: 10.1016/j.coldregions.2014.12.008

[22] Hotineanu A, Bouasker M, Aldaood A, Al-Mukhtar M. Effect of freeze–thaw cycling on the mechanical properties of lime-stabilized expansive clays. Cold Regions Sci Technol. 2015;119:151–7. doi: 10.1016/j.coldregions.2015.08.008

[23] Yang QW, Pei XJ, Huang RQ. Research on the effect of freeze and thaw cycles on the property and damage mechanism of M-CMC stabilized soil. Chinese J Rock Mechanics Eng. 2019;38(S1):3102–13.

[24] Li L, Shao W, Li Y, Cetin B. Effects of climatic factors on mechanical properties of cement and fiber reinforced clays. Geotech Geol Eng. 2014;33(3):537–48. doi: 10.1007/s10706-014-9838-4

[25] Fateh S, Mansourkiaei Y, Shalchian MM, Arabani M, Payan M, Ranjbar PZ. A comparison of temperature and freeze-thaw effects on high-swelling and low-swelling soils stabilized with xanthan gum. Results in Engineering. 2025;25:103719. doi: 10.1016/j.rineng.2024.103719

[26] Hatefi MH, Arabani M, Payan M, Zanganeh Ranjbar P. The influence of volcanic ash (VA) on the mechanical properties and freeze-thaw durability of lime kiln dust (LKD)-stabilized kaolin clayey soil. Results Eng. 2024;24:103077. doi: 10.1016/j.rineng.2024.103077

[27] Bell FG. Lime stabilization of clay minerals and soils. Eng Geol. 1996;42(1):71–80.

[28] Boardman DI, Glendinning S, Rogers CDF. Development of stabilisation and solidification in lime-clay mixes. Geotechnique. 2001;51(6):533–43.

[29] Tremblay H, Leroueil S, Locat J. Mechanical improvement and vertical yield stress prediction of clayey soils from eastern Canada treated with lime or cement. Can Geotech J. 2001;38(3):567–79. doi: 10.1139/t00-119

[30] Lu WX, Yang YH. Experimental study on lime improvement of loess subgrade filler. Low Temperature Architecture Technol. 2013;35(10):15–7.

[31] Zhao QH, Liang BM, X N. Experiment study of filling material on lime-improved loess for high-speed railway embankment. J Railway Sci Eng. 2005;2(6):53–7.

[32] Liu SY, Zhang T, Cai GJ. Research on technology and engineering application of silt subgrade solidified by lignin-based industrial by-product. China J Highway and Transport. 2018;31(3):1–11.

[33] Zhang T, Cai GJ, Liu SY. Research on stabilization microcosmic mechanism of lignin based industrial byproduct treated subgrade silt. Rock and Soil Mechanics. 2016;37(6):1665–72.

[34] Liu ZZ, Wang Q, Zhong XM. Water holding capacity and water stability of lignin-modified loess. Chinese J Rock Mechanics and Engineering. 2020;39(12):2582–92.

[35] Hou X, Ma W, Li GY. Influence of lignosulfonate on mechanical properties of Lanzhou loess. Rock and Soil Mechanics. 2017;38(Suppl 2):18–26.

[36] Gao ZN, Wang Q, Zhao CC. Effect of mixing method of sample preparation on the strength of loess improved by lignin fiber. World Earthquake Eng. 2021;43(4):930–4.

[37] Zhong XM, Liu W, Liu ZZ. Effect of different sample preparation methods on mechanical properties of lignin improved loess. World Earthquake Engineering. 2020;36(1):197–204.

[38] Varsha B, Moghal AAB, Rehman AU, Chittoori BCS. Shear, consolidation characteristics and carbon footprint analysis of clayey soil blended with calcium lignosulphonate and granite sand for earthen dam application. Sustainability. 2023;15(7):6117. doi: 10.3390/su15076117

[39] Li JH. Study on consolidation effect of soft soil layer not penetrated by plastic drainage belt by vacuum pre-compression method. Construction Technology. 2017;46(S1):221–4.

[40] Alielahi H, Maleki M, Mehrshahi K. Performance evaluation of a surcharge preloading project based on back-analysis of field monitoring and numerical assessment. Arab J Geosci. 2021;14(21). doi: 10.1007/s12517-021-08529-7

[41] Fakharian K, Mehdizadeh A. Investigation of field instrumentation in a preloading project. Proceedings of the Institution of Civil Engineers - Geotechnical Engineering. 2015;168(1):87–98. doi: 10.1680/geng.13.00018

[42] Luo L, Wang X, Xue C, Wang D, Lian B. Laboratory experiments and numerical simulation study of composite-material-modified loess improving high-speed railway subgrade. Polymers (Basel). 2022;14(15):3215. doi: 10.3390/polym14153215 35956729 PMC9371065

[43] Auersch L. Dynamic behavior of slab tracks on homogeneous and layered soils and the reduction of ground vibration by floating slab tracks. J Eng Mech. 2012;138(8):923–33. doi: 10.1061/(asce)em.1943-7889.0000407

[44] Standard Writing Group of the People’s Republic of China. Standard for geotechnical test methods: GB/T 50123—2019. Beijing: China Planning Press. 2019.

[45] Xu P, Lin Q, Fang L. Study on the Mechanical Properties of Loess Improved by Lignosulfonate and Its Mechanism Analysis and Prospects. Applied Sciences. 2022;12(19):9843. doi: 10.3390/app12199843

[46] Zhong XM, Liu W, Liu ZZ. Influence of different sample preparation methods on mechanical properties of lignin-amended loess. World Earthquake Eng. 2020;36(1):197–204.

[47] Ministry of Water Resources of the People’s Republic of China. GB/T 50123-2019; Standard for Geotechnical Testing Method. Beijing, China: China Standard Press. 2019.

[48] Jha AK, Sivapullaiah PV. Mechanism of improvement in the strength and volume change behavior of lime stabilized soil. Engineering Geology. 2015;198:53–64. doi: 10.1016/j.enggeo.2015.08.020

[49] Kavak A, Baykal G. Long-term behavior of lime-stabilized kaolinite clay. Environ Earth Sci. 2011;66(7):1943–55. doi: 10.1007/s12665-011-1419-8

[50] Cai G, Zhang T, Liu S, Li J, Jie D. Stabilization Mechanism and Effect Evaluation of Stabilized Silt with Lignin Based on Laboratory Data. Marine Georesour Geotechnol. 2014;34(4):331–40. doi: 10.1080/1064119x.2014.966217

[51] Tingle JS, Santoni RL. Stabilization of Clay Soils with Nontraditional Additives. Transportation Research Record: Journal of the Transportation Research Board. 2003;1819(1):72–84. doi: 10.3141/1819b-10

[52] Zhang T, Liu S, Cai G, Puppala AJ. Experimental investigation of thermal and mechanical properties of lignin treated silt. Eng Geol. 2015;196:1–11. doi: 10.1016/j.enggeo.2015.07.003

[53] Nuruddin M, Moghal AAB, Rasheed R. Coupled effect of ground granulated blast slag and bagasse fibers to enhance the compressive strength and durability characteristics of expansive soil. Geotechnical Frontiers. 2025; 323–31.

[54] Standard of Railway Subgrade Design. Beijing, China: China Railway Press. 2016.

[55] Xie X, Wang LY, Deng LJ. Study on the microscopic mechanism of the loess improved by quicklime. Coal Geol Exploration. 2021;49(6):193–9.

[56] Zhang Y, He H, Zeng ZY. Comparison of strength characteristics of fly ash-lime improved loess and compacted loess. Science Technol Eng. 2021;21(8):3265–73.

[57] Amulya G, Moghal AAB, Almajed A. Sustainable binary blending for low-volume roads-reliability-based design approach and carbon footprint analysis. Materials (Basel). 2023;16(5):2065. doi: 10.3390/ma16052065 36903183 PMC10003833

